# Socio-demographic factors influencing obesity among ever-married Jordanian women of reproductive age: insights from the 2023 Jordan demographic and health survey

**DOI:** 10.1186/s41043-026-01273-2

**Published:** 2026-03-10

**Authors:** Amr Ahmed Aly Ibrahim, Sara Hosny El-Farargy, Shadi Isac, Moaz Yasser Darwish, Mahmoud Shaaban Abdelgalil

**Affiliations:** 1 Faculty of Medicine, Monoufiya University, Monoufiya, Egypt; 2 Faculty of Medicine, Benha University, Benha, Egypt; 3 Faculty of Medicine, Ain-shams University, Cairo, Egypt; 4 Faculty of Medicine, Fayoum University, Fayoum, Egypt; 5 Research Insights Arab Network, Cairo, Egypt

**Keywords:** Obesity, Jordanian women, Jordan demographic and health survey (JDHS), Reproductive age

## Abstract

**Background:**

Obesity prevalence has been rising globally, including in Jordan. This study seeks to examine the socio-demographic factors associated with obesity among adult ever-married women in Jordan, utilizing the most recent data from the 2023 JPFHS.

**Methods:**

This cross-sectional study analyzed data from the 2023 JDHS, encompassing a representative sample of 4,048 Jordanian women aged 15–49. Socioeconomic variables examined included age, education level, wealth index, urban or rural residence, and media consumption habits (television, radio, magazines/newspapers, internet usage) along with smoking status. Multivariate logistic regression was employed to determine the associations between these factors and obesity risk.

**Results:**

Of the 4,048 married women included in the analysis, 1,697 (41.9%) had a normal BMI, while 2,351 (58.1%) were classified as obese. Multivariate analysis revealed that increasing age (45–49 years: AOR 20.93, 95% CI 13.40–32.70), daily internet use (AOR 1.33, 95% CI 1.02–1.74), listening to the radio less than once a week (AOR 1.40, 95% CI 1.03–1.91), and residing in Karak (AOR 2.13, 95% CI 1.34–3.38) or Ajloun (AOR 1.75, 95% CI 1.12–2.72) were significantly associated with higher odds of obesity. Conversely, reading newspapers or magazines at least once a week and daily cigarette smoking were linked to a reduced risk of obesity. No significant associations were observed between obesity and place of residence, wealth index, educational level, television viewing habits, or residence in other governorates.

**Conclusion:**

With obesity rates continuing to rise, targeted health programs for Jordanian women of reproductive age are essential. National health initiatives should focus on promoting healthy lifestyle habits, addressing regional disparities, and encouraging balanced media consumption to mitigate obesity risk. Region-specific prevention and awareness campaigns are also vital for effective intervention.

## Introduction

Obesity has emerged as a rapidly growing global health crisis, particularly in low- and middle-income countries undergoing socioeconomic transitions [1]. It is characterized by excessive fat accumulation, which poses significant health risks, particularly with the rising incidence of non-communicable diseases [1, 2]. The World Health Organization (WHO) now classifies obesity as a pandemic, with over 2 billion adults worldwide being overweight, and 677.6 million of them classified as obese [3, 4].

Women, in particular, face high rates of obesity, comprising 393.5 million, which places them at increased risk for numerous health issues, including cardiovascular diseases, type 2 diabetes, and certain cancers [3, 5]. Additionally, obesity among women of reproductive age is linked to challenges with conception, an elevated risk of miscarriage, and various complications during pregnancy, labor, and postpartum, contributing to higher rates of maternal morbidity and mortality [6–8]. The COVID-19 pandemic has further complicated the landscape, amplified sedentary lifestyles and increased the demand for weight-loss surgeries, such as gastric sleeve procedures, as individuals struggle to manage weight in the face of lifestyle changes and limited access to physical activity [9].

Jordan, like other countries in the Middle East, has witnessed a significant rise in obesity rates, especially among women [10]. With 43.1% of Jordanian females considered obese compared to 28.2% of males, the trend has been steadily increasing at an annual rate of 1.38% over the last two decades [11]. According to the Global Obesity Observatory, the prevalence of obesity among females is projected to reach 77.54% by 2060. Additionally, the economic burden of obesity is expected to expand, with associated costs shifting from USD 850.48 million in 2019 to USD 8.97 billion by 2026 [12]. The regional obesity crisis has been driven by several factors, including dietary shifts, urbanization, changing marital norms, and a higher intake of processed foods and sugary beverages [10].

A study based on data from the 2009 Jordan Population and Family Health Survey (JPFHS) identified several factors associated with obesity over time, including age, residence in southern regions of Jordan, early marriage, parity, wealth status, and smoking [13]. Similarly, an analysis of data from the 2017/18 JPFHS, which included 4,226 Jordanian women of reproductive age, highlighted additional obesity-related factors [14]. The study found that increasing age and residing in Tafilah were significantly associated with higher odds of obesity, while belonging to the wealthiest category, living in Maan or Aqaba, and daily smoking were linked to reduced odds of obesity [14].

Despite previous research [13, 14], obesity remains a significant and growing public health concern in Jordan. The rapidly evolving socio-economic, cultural, and lifestyle patterns in the region highlight the need for updated studies to reflect current trends. This study seeks to examine the socio-demographic factors associated with obesity among adult ever-married women in Jordan, utilizing the most recent data from the 2023 JPFHS.

## Methods

### Data collection

Our cross-sectional study utilized data from 2023 JPFHS. The survey initially recruited a representative sample of 4,048 eligible women aged 15–49 from all 12 governorates in Jordan. Key data collected included anthropometric measurements, particularly body mass index (BMI), alongside socioeconomic and behavioral factors.

### Inclusion criteria

We included data from ever-married women aged 20–49 years with a reported BMI (kg/m²) measured in accordance with the guidelines of the Centers for Disease Control and Prevention [15].

### Exclusion criteria

Women with missing BMI measurements were excluded to maintain data completeness. The analysis was restricted to ever-married women; therefore, women younger than 20 years were excluded because they fall outside the study’s target population. In addition, women classified as underweight (BMI < 18.5 kg/m²) or overweight (BMI 25–29.9 kg/m²) were excluded to enable a focused comparison between normal weight and obesity. This approach allowed a clearer assessment of socio-demographic and behavioral factors specifically associated with obesity-related BMI status.

### Included variables

We studied some socioeconomic and behavioral variables including Age (divided into five-year groups: 20–24, 25–29, 30–34, 35–39, 40–44 and 45–49 years of age), type of residence (rural or urban), governorate of residence (Mafraq, Ajloun, Amman, Zarqa, Karak, Tafiela, Madaba, Aqaba, Irbid, Balqa, Jarash, and Ma’an), wealth index as classified by the DHS survey (richest, richer, middle, poorer and poorest), educational level (higher education, secondary education, primary education and no education), and frequency of listening to the radio, watching television, reading magazines or newspapers or frequency of internet usage (at least once a week, less than once a week and not at all). We also added the frequency of smoking (everyday smoking, some days smoking, and does not smoke).

### Statistical analysis

All variables were coded according to DHS standard definitions prior to analysis [16]. Body mass index (BMI) was categorized into normal weight (18.5–24.9 kg/m²) and obesity (≥ 30.0 kg/m²), with normal weight used as the reference category. Socio-demographic and behavioral variables were included as categorical independent variables as described above. Descriptive analyses were conducted using weighted frequencies and percentages to summarize participants’ characteristics. A multivariable logistic regression model was fitted directly to examine the independent associations between each covariate and obesity while controlling for all other variables. Results were reported as adjusted odds ratios (AORs) with 95% confidence intervals (CIs). All analyses accounted for the DHS sampling weights to reflect the complex survey design, and statistical significance was set at *p* < 0.05.

## Results

Data was collected from 4,048 Jordanian women aged 20–49, with 41.9% of the sample having normal BMI and 58.1% being obese (Table 1).

**Table 1:** Characteristics of included women by BMI category (Normal weight vs. Obese), JPFHS 2023

		BMI			
		Normal		Obese	
		Count	Column N %	Count	Column N %
**Age in 5-year groups**	20–24	238	14.0%	75	3.2%
	25–29	360	21.2%	203	8.6%
	30–34	404	23.8%	370	15.7%
	35–39	329	19.4%	481	20.4%
	40–44	219	12.9%	513	21.8%
	45–49	147	8.6%	709	30.1%
**Highest educational level**	No education	44	2.5%	58	2.4%
	Primary	100	5.7%	224	9.5%
	Secondary	876	50.1%	1456	61.5%
	Higher	729	41.7%	631	26.6%
**Wealth index combined**	Poorest	407	23.3%	539	22.8%
	Poorer	342	19.6%	545	23.0%
	Middle	393	22.5%	494	20.9%
	Richer	272	15.5%	480	20.3%
	Richest	335	19.2%	309	13.1%
**Type of place of residence**	Urban	1589	90.8%	2159	91.1%
	Rural	160	9.2%	210	8.9%
**Frequency of reading newspaper or magazine**	Not at all	1290	73.8%	1852	78.2%
	Less than once a week	200	11.4%	246	10.4%
	At least once a week	259	14.8%	270	11.4%
	Almost every day	0	0.0%	0	0.0%
**Frequency of listening to radio**	Not at all	1236	70.7%	1661	70.1%
	Less than once a week	227	13.0%	330	13.9%
	At least once a week	286	16.3%	378	15.9%
	Almost every day	0	0.0%	0	0.0%
**Frequency of watching television**	Not at all	278	15.9%	368	15.5%
	Less than once a week	346	19.8%	438	18.5%
	At least once a week	1126	64.3%	1563	66.0%
	Almost every day	0	0.0%	0	0.0%
**Frequency of using internet last month**	Not at all	430	24.6%	480	20.3%
	Less than once a week	25	1.4%	40	1.7%
	At least once a week	45	2.6%	86	3.6%
	Almost every day	1249	71.4%	1764	74.5%
**Frequency smokes cigarettes**	Does not smoke	1568	89.6%	2171	91.7%
	Every day	158	9.0%	137	5.8%
	Some days	24	1.3%	60	2.5%
**Governorate**	Central region				
	Amman	891	50.9%	988	41.7%
	Balqa	99	5.7%	154	6.5%
	Zarqa	220	12.6%	376	15.9%
	Madaba	26	1.5%	51	2.2%
	Northern region				
	Irbid	297	17.0%	458	19.3%
	Mafraq	71	4.1%	84	3.5%
	Jarash	35	2.0%	62	2.6%
	Ajloun	23	1.3%	46	2.0%
	Southern Region				
	Karak	26	1.5%	65	2.8%
	Tafiela	13	0.7%	20	0.9%
	Ma’an	23	1.3%	25	1.1%
	Aqaba	25	1.4%	37	1.6%

The result showed that the highest proportion of obese participants was from the 35 to 49 years age group representing 72.3% of total obese women with the highest proportion between 45 and 49 representing 30.1%. However, 45% of normal BMI females were 25–34 years old with the highest proportion between 30 and 34.

Among women with normal BMI, approximately half (50.1%) had completed their secondary and 41.7% had a higher learning degree. On the other hand, of the obese population, 61.5% of them reached the secondary educational level and 26.6% had a higher learning degree.

Regarding residence, about 90.8% of the normal BMI and 91.1% of the obese group lived in urban. Most of the normal BMI group and the obese group lived in: Amman with 50.9% in the normal BMI group and 41.7% in the obese group, Irbid with 17% in the normal BMI group and 19.3% in the obese group, and Zarqa with 12.6% in the normal BMI group and 15.9% in the obese group.

Obesity was more predominant in the poorer group 23% followed by the poorest, middle, richer, and richest (22.8%, 20.9%, 20.3%, and 13.1% respectively). On the other hand, the normal BMI was predominant in the poorest group 23.3% followed by middle, poorer, richest, and richer (22.5%, 19.6%, 19.2%, and 15.5%, respectively).

Most of the participants in both groups did not read magazines or newspapers at all with 73.8% in the normal BMI group and 78.2% in the obese group. Additionally, 11.4% in the normal BMI group and 10.4% in the obese group did less than once a week, while 14.8% in the normal BMI group and 11.4% in the obese group did at least once a week.

Most of the participants in both groups did not listen to the radio at all with 70.7% in the normal BMI group and 70.1% in the obese group. Additionally,13% in the normal BMI group and 13.9% in the obese group listened to the radio less than once a week while 16.3% in the normal BMI group and 15.9% in the obese group listened to the radio at least once a week.

64.3% of the normal BMI group and 66% of the obese group watched television at least once a week. In contrast,19.8% of the normal BMI group and 18.5% of the obese group did less than once a week, while 15.9% of the normal BMI group and 15.5% of the obese group didn’t watch at all.

The majority of the normal BMI group (71.4%) and the obese group (74.5%) used the internet last month almost every day, while 24.6% of the normal BMI group and 20.3% of the obese group didn’t use the internet at all. Regarding smoking, 89.6% of the normal BMI group and 91.7% of the obese group didn’t smoke.

In multivariable analysis **(**Table 2**)**, we found that the odds of obesity increased with increasing the women’s age, with the age group of 45–49 years conveying the highest OR (OR: 20.930; 95%CI: 13.396–32.702; *p* < 0.001) compared to the age group of 20–24 years.

**Table 2:** Sociodemographic and Economic Predictors of Obesity Among Included Women: Adjusted Odds Ratios (AORs) and 95% Confidence Intervals

**Parameters estimate**							
**Variables**		**B**	**Standard error**	**AOR**	**95% Confidence interval for AOR**		
					**Lower**	**Upper**	**P value**
**Age at 5 years**	**20-24**	**Reference**					
	**25-29**	0.723	0.218	2.061	1.342	3.164	**0.001**
	**30-34**	1.377	0.215	3.963	2.601	6.037	**< 0.001**
	**35-39**	1.874	0.215	6.517	4.274	9.938	**< 0.001**
	**40-44**	2.290	0.223	9.872	6.372	15.294	**< 0.001**
	**45-49**	3.041	0.227	20.930	13.396	32.702	**< 0.001**
**Highest Educational level**	**No Education**	**Reference**					
	**Primary**	0.483	0.394	1.621	0.748	3.514	0.220
	**Secondary**	0.082	0.341	1.086	0.555	2.122	0.810
	**Higher**	-0.512	0.350	0.599	0.302	1.190	0.143
**Governorate**	**Aqaba**	**Reference**					
	**Ma'an**	-0.131	0.228	0.877	0.561	1.372	0.565
	**Tafiela**	0.245	0.236	1.277	0.804	2.030	0.300
	**Karak**	0.755	0.236	2.128	1.340	3.378	**0.001**
	**Ajloun**	0.558	0.225	1.747	1.124	2.715	**0.013**
	**Jarash**	0.352	0.219	1.422	0.925	2.184	0.108
	**Mafraq**	0.004	0.248	1.004	0.617	1.634	0.987
	**Irbid**	0.129	0.203	1.138	0.764	1.694	0.524
	**Madaba**	0.379	0.233	1.461	0.926	2.307	0.103
	**Zarqa**	0.372	0.199	1.451	0.981	2.145	0.062
	**Balqa**	0.236	0.200	1.266	0.855	1.876	0.239
	**Amman**	-0.235	0.194	0.791	0.540	1.158	0.228
**Wealth index**	**Poorest**	**Reference**					
	**Poorer**	0.230	0.173	1.259	0.897	1.767	0.183
	**middle**	0.045	0.169	1.046	0.751	1.456	0.791
	**Richer**	0.233	0.198	1.262	0.856	1.861	0.240
	**Richest**	-0.314	0.253	0.731	0.445	1.200	0.215
**Type of place residence**	**Urban**	**Reference**					
	**Rural**	-0.215	0.129	0.807	0.626	1.040	0.098
**Frequency of using Internet last month**	**Not at all**	**Reference**					
	**Less than once a week**	0.316	0.473	1.372	0.543	3.470	0.504
	**At least once a week**	0.518	0.296	1.678	0.939	3.001	0.081
	**Almost everyday**	0.285	0.137	1.330	1.016	1.741	**0.038**
**Frequency of watching Television**	**Not at all**	**Reference**					
	**Less than once a week**	-0.067	0.191	0.936	0.643	1.360	0.727
	**At least once a week**	0.103	0.148	1.109	0.829	1.484	0.487
**Frequency of listening to Radio**	**Not at all**	**Reference**					
	**Less than once a week**	0.335	0.158	1.398	1.026	1.906	**0.034**
	**At least once a week**	0.185	0.162	1.204	0.876	1.655	0.253
**Frequency of reading newspaper or magazine**	**Not at all**	**Reference**					
	**Less than once a week**	-0.345	0.233	0.708	0.448	1.119	0.139
	**At least once a week**	-0.480	0.146	0.619	0.465	0.823	**0.001**
**Frequency smokes cigarettes**	**Does not smoke**	**Reference**					
	**Everyday**	-0.669	0.189	0.512	0.354	0.742	**<0.001**
	**Somedays**	0.350	0.506	1.420	0.526	3.833	0.489

AOR: adjusted odds ratio

Regarding media consumption, women who listened to the radio less than once a week were more likely to be obese (OR: 1.398; 95% CI: 1.026–1.906; *p* = 0.034) than those who never listened to the radio. However, there was no significant association between obesity and women who listened to the radio at least once a week (OR: 1.204; 95% CI: 0.876–1.655; *p* = 0.253).

Also, we found that women who used the internet almost every day in the last month were more likely to be obese (OR: 1.330; 95%CI: 1.016–1.741; *p* = 0.038) than those who didn’t use it. In contrast, women who used the internet less than once a week (OR: 1.372; 95%CI: 0.543–3.470; *p* = 0.504) and at least once a week (OR: 1.678; 95%CI: 0.939–3.001; *p* = 0.081) showed no significant association with obesity.

Regarding the frequency of reading newspapers or magazines, women who read at least once a week (OR: 0.619; 95%CI: 0.465–0.823; *p* = 0.001) were associated with a lower risk of obesity than those who didn’t read. However, reading less than once a week showed no significant association with obesity (OR: 0.708; 95%CI 0.448–1.119; *p* = 0.139).

In terms of Smoking, we found that everyday smoking was significantly associated with lower odds of obesity (OR: 0.512; 95%CI: 0.354–0.742; *p* < 0.001) while some days smoking was not significantly associated with obesity (OR: 1.420; 95%CI: 0.526–3.833; *p* = 0.489).

Geographical location also played a role, as living in Karak and Ajloun was significantly associated with higher odds of obesity (OR: 2.128; 95%CI: 1.340–3.378; *p* = 0.001), (OR: 1.747; 95%CI: 1.124–2.715; *p*=0.013) respectively.

On the other hand, we found no significant association between obesity and the education level, wealth index, type of place of residence, and frequency of watching television (*p* > 0.05).

## Discussion

To the best of our knowledge, this study is the first to explore the socio-demographic factors influencing obesity among ever-married adult women in Jordan, using data from the 2023 JDHS.

The prevalence of obesity in Jordan has risen sharply in recent years. In 2009, 38.8% of individuals were classified as obese [13], increasing to 51.3% by 2017/18 [14], and reaching 58.1% in 2023, as observed in this study. This upward trend may be partly driven by significant lifestyle changes and unhealthy eating behaviors following the COVID-19 pandemic [17], as lockdowns, distance learning, and remote work led to more sedentary routines.

Our analysis identified a significant association between age and obesity, revealing that older women had considerably higher obesity rates. Specifically, women aged 44–49 were 20.9 times more likely to be obese compared to those aged 20–24. These findings are consistent with previous studies conducted in Jordan, including Al Nsour et al. 2008 [13], Bustami et al. 2021 [18], and Shaaban Abdelgalil et al. 2024 [14] as well as studies by Pengpid et al. in Iraq 2015 [19], and Chamieh et al. in Lebanon 2015 [20], all of which reported a significantly higher prevalence of obesity among older women.

This trend is largely due to hormonal and psychological changes associated with aging. As women approach menopause, declining estrogen and increased androgens lead to muscle loss, increased abdominal fat, and altered body composition. Coupled with a sedentary lifestyle, these changes reduce energy expenditure and basal metabolic rate, increasing obesity risk [21, 22]. Additionally, psychological changes like increased stress, mood fluctuations, and anxiety often trigger emotional or stress-induced eating, further contributing to obesity risk [23].

The analysis of obesity odds across regions revealed significant variations. Women in Karak, located in the southern region, and Ajloun, in the northern region, had significantly higher odds of obesity compared to those in Aqaba in the southern region. These findings differ from those of Shaaban Abdelgalil et al. 2024 in Jordan [14], which reported no significant differences in obesity odds between the central and northern governorates. However, the study noted that in the southern governorates, living in Tafilah in southern region was associated with an increased likelihood of obesity, whereas residing in Maan or Aqaba in southern region was linked to a decreased likelihood [14].

The regional variations in obesity odds among Jordanian women can be explained by a combination of factors. Socioeconomic inequalities may restrict access to nutritious food options and quality healthcare, while cultural and lifestyle practices, such as consuming traditional high-calorie diets and engaging in minimal physical activity, further elevate obesity risks in certain areas [24, 25]. Additionally, regions with lower levels of urbanization often lack the necessary infrastructure, such as parks and recreational facilities, to encourage physical activity, leading to more sedentary lifestyles [26]. Differences in healthcare access and public awareness also contribute, as areas with limited healthcare resources may fall short in providing adequate education and interventions for obesity prevention and management [24, 27].

Our study found that certain media habits were linked to obesity. Women who listened to the radio less than once a week had higher odds of obesity compared to those who never listened. This finding is consistent with El-Qushayri et al. 2023 in Egypt [28]. It is possible that individuals who listen to the radio may engage in less physical activity, leading to a more sedentary lifestyle, which is associated with obesity [29].

Furthermore, regular internet use was associated with higher odds of obesity compared to individuals who did not use the internet at all. This suggests that frequent internet use may limit the time available for physical activity [29, 30]. A meta-analysis by Aghasi et al. found a linear dose-response relationship, indicating that each additional hour of internet use per day was associated with an 8% increase in the odds of overweight and obesity [31].

Conversely, our study found no association between watching television and obesity, which contrasts with studies in countries such as Ghana, Bangladesh, and Myanmar, where higher television consumption has been linked to obesity due to prolonged sedentary behaviors and exposure to unhealthy food advertisements [32–34]. Our study suggests that appropriate actions should be taken through both traditional and social media, targeting obese Jordanian women to raise awareness about the negative effects of reduced physical activity, excessive radio listening, and frequent internet use.

Smoking was significantly associated with lower odds of obesity among daily smokers in our sample. This finding aligns with previous studies by Al Nsour et al. 2009 in Jordan [13], Shaaban Abdelgalil et al. 2024 in Jordan [14], Watanabe et al. 2016 in Japan [35] and Dare et al. 2015 in the UK [36]. Some studies suggest that nicotine may contribute to appetite suppression, which could explain the link between smoking and a reduced risk of obesity [37]. However, while smoking may be associated with lower obesity risk, it also carries significant health risks, including higher rates of central adiposity, cardiovascular disease, and cancer [37, 38].

Unlike some studies conducted in other countries, our findings did not show a significant association between education level and obesity among women, aligning with the study by Shaaban Abdelgalil et al. 2024 in Jordan [14]. However, some studies have suggested that women with secondary education experience higher obesity rates compared to those with higher education. Research has often linked higher education to improved health outcomes, largely due to greater health literacy and socioeconomic advantages [39–41]. Nevertheless, variations in findings across countries, including Ghana, Bangladesh, and Ethiopia, highlight the diverse influence of social determinants of health and the differing effects of education on lifestyle and diet [42–45].

Our study found no significant association between place of residence and obesity among Jordanian women, consistent with the findings of Shaaban Abdelgalil et al. 2024 in Jordan [14]. This contrasts with findings from some low- and middle-income countries, where urban living is often associated with higher obesity rates [39, 46–48]. In our sample, both urban and rural areas exhibited similar obesity prevalence, likely due to shared dietary patterns and lifestyle similarities within Jordan’s relatively compact urban-rural structure.

Our analysis did not find a significant association between wealth index and obesity, which aligns with the study by Shaaban Abdelgalil et al. 2024 in Jordan [14]. However, wealth index is still a relevant factor, as obesity rates were higher among women in the poorer wealth quintiles, contrasting with findings in other countries where obesity is often more prevalent among the affluent [42, 49–51]. In Jordan, socioeconomic factors may influence dietary choices, with lower-income individuals more likely to choose cheaper, calorie-dense foods over more nutritious alternatives. Additionally, cultural perceptions that associate larger body sizes with well-being may also contribute to obesity patterns among wealthier women in some Middle Eastern countries [47, 50, 52].

### Limitations and recommendations

Our study utilized data from the 2023 JPFHS, focusing on ever-married women aged 20–49 years, but several limitations must be acknowledged. First, as a cross-sectional study, it cannot establish causal relationships between obesity and associated factors. Second, our analysis excluded specific groups, such as women younger than 20 years, and single women, which may have introduced selection bias and limited the generalizability of our findings. Third, the exclusion of comorbidities from the dataset, despite their potential role in influencing obesity, further limits the scope of our analysis. Additionally, the reliance on self-reported media consumption habits and internet usage may be subject to recall bias, potentially affecting the accuracy of the associations observed.

Despite these limitations, our findings offer actionable insights into obesity determinants among Jordanian women. We recommend future research to include broader age groups and examine the role of comorbidities to provide a more comprehensive understanding of obesity risk factors. Longitudinal studies are particularly needed to establish causality and capture temporal trends in obesity and its predictors. From a policy perspective, targeted interventions addressing age-related increases in obesity are essential. These interventions should focus on promoting healthy lifestyles, particularly among women with frequent internet use or irregular physical activity. Additionally, regional disparities, such as higher obesity odds in Karak and Ajloun, call for tailored, location-specific programs. National initiatives emphasizing nutrition education, lifestyle modifications, and community-based support could play a pivotal role in curbing obesity rates among Jordanian women.

## Conclusion

The current study identified key sociodemographic factors associated with obesity among adult ever-married women in Jordan. Older women, frequent internet users, those who listen to the radio less frequently, and residents of Karak and Ajloun were associated with higher obesity rates. Conversely, everyday smoking is linked to lower odds of obesity. Targeted interventions tailored for older women and regions with higher obesity rates is recommended to address the potential age and region disparities. Strategies to promote healthier lifestyles and encourage physical activity is necessary to combat modern lifestyle behaviors leading to obesity.

## Data Availability

Data is available upon request from ICF International’s website (https://dhsprogram.com/data/available-datasets.cfm).
